# Complex Coronary Hemodynamics - Simple Analog Modelling as an Educational Tool

**DOI:** 10.2174/1874431101711010012

**Published:** 2017-07-28

**Authors:** Gaurav R. Parikh, Elvis Peter, Nikolaos Kakouros

**Affiliations:** 1 Division of Cardiovascular Medicine, University of Massachusetts, 55 Lake Ave North, Worcester, MA, 01655. USA; 2 Department of Cardiology, Marshfield Clinic, Weston Center 3501 Cranberry Blvd, Weston, WI 54476, USA

**Keywords:** Coronary angiography, Myocardial fractional flow reserve, Computational modelling, Coronary hemodynamics, System equivalence, Educational tools

## Abstract

**Objective:**

Invasive coronary angiography remains the cornerstone for evaluation of coronary stenoses despite there being a poor correlation between luminal loss assessment by coronary luminography and myocardial ischemia. This is especially true for coronary lesions deemed moderate by visual assessment. Coronary pressure-derived fractional flow reserve (FFR) has emerged as the gold standard for the evaluation of hemodynamic significance of coronary artery stenosis, which is cost effective and leads to improved patient outcomes. There are, however, several limitations to the use of FFR including the evaluation of serial stenoses.

**Method:**

In this article, we discuss the electronic-hydraulic analogy and the utility of simple electrical modelling to mimic the coronary circulation and coronary stenoses. We exemplify the effect of tandem coronary lesions on the FFR by modelling of a patient with sequential disease segments and complex anatomy.

**Results:**

We believe that such computational modelling can serve as a powerful educational tool to help clinicians better understand the complexity of coronary hemodynamics and improve patient care.

## 1 INTRODUCTION

Invasive catheter-based coronary angiography remains the de facto standard for the evaluation of coronary anatomy. Nonetheless, luminal loss assessment as evaluated by angiography correlates poorly with myocardial ischemia. Coronary pressure-derived fractional flow reserve (FFR) has emerged as the gold standard for the invasive evaluation of the hemodynamic significance of coronary artery stenoses.

FFR is effectively an invasive stress test that can be performed in the catheterization laboratory. The coronary artery is engaged with a guide catheter and, after adequate anticoagulation, a 0.014 inch guidewire is introduced into the distal vessel past the coronary stenosis. The wire contains a pressure sensor that measures pressure distal (Pd) to the stenosis while a pressure transducer connected to the proximal end of the guide catheter measures the aortic pressure (Pa). Intravenous or intracoronary adenosine is used to achieve maximal hyperemia. The microvascular bed resistance, in the myocardium, is relatively low as it comprises multiple resistive vessels in parallel. Furthermore, at maximal vasodilatation, the microvascular resistance is minimal yielding a constant ratio between flow and pressure. Consequently, the effect of a stenosis on flow can then be evaluated by the simplified ratio of the pressures distal and proximal to the stenosis (Pd:Pa) [1].

Normal epicardiac coronary vessels have a negligible resistance and hence, in absence of coronary stenosis, there is no pressure drop in the epicardial coronary tree such that the ideal normal FFR will be 1 when measured at a distal vessel. Nonetheless, in the presence of coronary stenosis, the distal pressure drops as a function of the introduced epicardial resistance and flow though the vessel. The vessel resistivity is directly proportional to the lesion length and indirectly proportional to the lesion cross-sectional area with some contribution by lesion morphology that can affect flow dynamics and lead to separation losses [2-4]. The overall flow past the resistive lesion is determined by the pressure difference across the system (systemic aortic pressure to venous pressure) and the total resistance of the coronary system (epicardial resistance introduced by stenosis plus resistance of the subtended myocardial bed) [3]. In the setting of maximal hyperemia, an FFR < 0.75 has been strongly associated with inducible myocardial ischemia on non-invasive stress tests with a sensitivity of 88%, specificity of 100% [5]. Subsequent randomized clinical trials (DEFER, FAME and FAME II) have shown that FFR-guided percutaneous coronary intervention (PCI) is not only safe and but also cost effective in yielding improved patient outcomes [6-8].

Three important axioms of coronary physiology are that a) FFR is relatively independent of changes in hemodynamic conditions, heart rate, blood pressure and myocardial contractility, b) FFR is related to the total hyperemic blood flow that is dependent on the myocardial bed resistance which is affected by multiple factors such as viability of the subtended myocardial bed, and c) FFR is agnostic to the source of pressure delivery at the distal circulation; therefore, it takes into account collateral circulation or dual blood supply.

Nonetheless, there are several special scenarios where one should be cautious in interpreting FFR values such as acute coronary syndrome, decompensated heart failure or untreated sleep apnea patients who may have elevated right atrial pressures that should be measured and taken into consideration especially in a borderline negative FFR value [9,10].

FFR can be accurately used to assess the hemodynamic significance of isolated left main disease however presence of downstream stenosis in either the left anterior descending (LAD) or left circumflex (LCx) will reduce the hyperemic blood flow through the left main and may give a falsely negative FFR result [11]. Finally, a frequent scenario that merits particular consideration is the use of FFR in assessing serial stenoses in the same coronary artery as this makes interpretation of FFR values challenging. Although the individual contribution of each stenosis can be accurately measured, this requires the measurement of coronary occlusion pressures and hence balloon dilation of at least one coronary segment [12].

The understanding and application of basic fluid dynamics including the interaction of tandem lesions and the effect of flow on the pressure drop induced by a fixed resistance is critical in the clinical application of the FFR technique but can be difficult to conceptualize. We present herein a patient with a chronically occluded right coronary artery (RCA) as well as two serial stenoses in the left anterior descending artery and describe the complex hemodynamic challenge of such anatomy using a simplified electrical model.

## 2 METHOD

We retrospectively selected a patient with complex coronary disease that had undergone FFR evaluation and PCI entirely on clinical grounds. Clinical findings collected at the time of this coronary intervention were then used to define the accuracy of the simulation model as described below. Written informed consent was obtained from the patient. The Institutional Review Board (IRB) at our institute determined that the proposed activity met parameters defined by DHHS and FDA regulations and that further approval was not required.

An 80 year-old woman with prior history of coronary artery disease presented with progressive chest discomfort upon minimal exertion despite being on maximal medical therapy. Her cardiac risk factors included hypertension and dyslipidemia. She had prior coronary stent placement to her proximal LAD and second obtuse marginal (OM2) branch of the LCx 3 years earlier. Her presentation electrocardiogram showed normal sinus rhythm and nonspecific T-wave changes with negative Troponin I. Further evaluation with a coronary angiogram showed a short left main with minor irregularities, 60% stenosis of proximal LAD and 90% stenosis of distal LAD. She also had a 60% stenosis of the proximal left circumflex artery (LCx). Her OM2 stent was patent without any restenosis. She had previously known 100% right coronary artery (RCA) occlusion.

She underwent further hemodynamic assessment of her coronary stenoses with a pressure wire using a PrimeWire Prestige pressure sensing wire (Volcano Corp., San Diego, CA). Maximal hyperemia was achieved with intravenous adenosine infusion at 180 mcg/kg/min. The FFR was assessed in the mid LAD and found to be 0.83, albeit with impaired distal vessel runoff due to the distal LAD stenosis.

The patient underwent successful treatment of the 90% distal LAD lesion with a 2.25 x 14 mm zotarolimus drug-eluting stent that was post-dilated using a 2.5 mm non-compliant balloon. A subsequent hemodynamic assessment of the proximal and mid LAD stenoses under maximal hyperemia showed the FFR in the mid-LAD to be now significant at 0.74. Consequently the proximal LAD lesion was treated with a 3.0 x 12 mm Zotarolimus drug eluting stent. A final FFR measurement in the LAD normalized at 0.96.

The coronary circulation can be considered as comprising of two distinct compartments: the epicardial “conductance vessels” that, under normal circumstances, present no significant resistance to the flow of blood and the small (<400μm diameter) arteries or “resistive vessels”. Normally, there is a constant correlation between the luminal cross-sectional area of a normal coronary artery at a point along its length and the mass of myocardium subtended by the artery beyond this point. Consequently, flow in an artery and downstream resistance depend on the amount of myocardium perfused. Also, as the normal epicardial vessels present no resistance, the pressure in the distal parts of the epicardial tree should normally match central aortic pressure.

The resistance, inductance and capacitance of the epicardial vessels in our model were modeled using resistors, inductors and capacitors. Nonetheless, the values of inductance and capacitance are minimal and the epicardial “conductance vessels” can be effectively modeled as ideal conductors (wires) with little intrinsic resistance to flow.

The subtended myocardium and its resistive vessels are represented by ideal resistors. As resistive vessels are in parallel, their total resistance is inversely proportional to their number. With the number of resistive vessels per mass of myocardium considered constant, the overall downstream resistance is, in turn, inversely related to the mass of myocardium. One of the most widely accepted and used scoring systems describing the relative myocardium subtended by each coronary artery is the Leaman score [13], based on pathological study measurements of relative epicardial vessel flow contribution [14]. In the right dominant coronary system, the right coronary artery (RCA) supplies 16% and the left main stem (LMS) 84% of the left ventricular (LV) mass. Two-thirds of the LMS flow is directed down the left anterior descending artery (LAD) and a third towards the left circumflex artery (LCx). In a left-dominant system, the RCA contribution is taken up by the LCx such that 58% of the total LMS flow goes to the LAD and 42% to the LCx. The value of the downstream resistive vessels in the present electrical model is based on the inverse of the Leaman weighing scores for a right-dominant coronary system; they are set at 30Ω for the RCA, 20Ω for the LCx, and 10Ω for the LAD myocardial bed.

The resistive values of the modelled epicardial stenoses are based on previous experimental work of fitting hydraulic-electrical analogous models to complex coronary anatomy [15]. For simplicity, a uniform input voltage of 100V is applied such that voltages down the simulated coronary tree can be easily and directly read as percentages of the input voltage (*viz*. central aortic pressure). Electrical models of the entire heart along with the pulmonary and systemic vasculature were also developed but in this case distract from the intended simplicity of the model for educational and demonstration purposes [16]. Similarly, diagonal branches of the LAD and obtuse marginal branches of the circumflex were excluded for clarity of demonstrating the serial lesion FFR principle.

The coronary angiogram, in Fig. (**1**), shows the proximal angiographically moderate LAD stenosis as well as the severe distal LAD stenosis, moderate LCx stenosis and collaterals to the occluded RCA. The corresponding final simplified electrical model, developed from the anatomy using the principles described above, is shown in Fig. (**2**). The model was built onto an online analog circuit simulator (CircuitLab, Inc., Mountain View, CA). Arterial “pressure” of 100mmHg was set at a voltage of 100V delivered by a voltage source, whereas a venous “pressure” of 5mmHg was simulated by an outflow voltage of 5V. Voltmeters were placed to display voltages at the mid and distal LAD as well as the distal LCx and RCA during simulation. The current flow at each distal resistor was also monitored to simulate blood flow to each myocardial bed.

The resistance of the angiographically near normal LMS was modelled at a minimal 10mΩ. The lesion of the moderate proximal LCx stenosis was set at a fixed 750mΩ, whereas the totally occluded proximal RCA was assigned an effectively occlusive 10kΩ.

Simulation was run at baseline settings to emulate the patient’s presenting coronary state (Sim.1; Table **1**). The resistance of the LAD2 resistor, simulating the distal LAD lesion, was then minimized to simulate PCI to this stenosis (Sim. 2; Table **1**). The resistance of the LAD1 resistor, simulating the proximal LAD stenosis, was then reduced to 0.1Ω to simulate effective PCI. A theoretical condition of PCI to the proximal LAD stenosis but not the distal stenosis was also modeled.

## RESULTS

The electrical model findings at baseline are consistent with the clinical findings of moderately reduced pressures in the mid LAD with FFR of 0.83 between the two lesions Table (**1**-sim. 1: 0.83). The most notable finding of the modelling is the demonstration of an increased voltage drop across the proximal resistance (LAD1) after reduction in the distal resistance (LAD2), commensurate with the clinical findings of lower mid LAD FFR after PCI to the distal LAD stenosis Table (**1** – sim. 2). It is evident from the model that this increased voltage (viz. pressure) drop at the proximal LAD is due to an increased flow; the current increased from 3.1A at baseline to 6.7A after abolishing the distal vessel resistance.

A notable finding from the simulation is that after reduction of the distal LAD resistance (LAD2) there is reduced voltage at the distal RCA part of the circuit (0.72 to 0.63). The simulation thus predicts that PCI to the distal LAD stenosis would result in further lowering of pressure at the distal RCA coronary and consequently reduced flow (2.23A at baseline to 1.94A). During coronary intervention to the LAD, anterolateral electrocardiographic (ECG) leads were being monitored but the catheterization laboratory system recorded additional leads in full disclosure mode that were retrieved to test this hypothesis Fig. (**3**). Interestingly, these did confirm worsening ST depression in the inferior leads, corresponding to the RCA territory, after PCI to the distal LAD lesion. This is suggestive of worsening ischemia in the RCA territory due to a coronary ‘steal’ phenomenon as predicted by the modelling [1, 17]. Prior studies have shown this “coronary steal” phenomenon to occur in about 40% of patients with chronic total occlusion due to maximally increased conductance at baseline of the myocardial bed subtended by the occluded vessel secondary to ischemic hyperemia [18]. The ischemic changes resolved after PCI to the proximal LAD, again consistent with the modelling prediction of improved flow in the distal RCA through enhanced collateral flow.

Finally, the theoretical modelling of intervention to the proximal LAD stenosis alone Table (**1** – sim. 4) shows that this would still result in low pressure delivery in the distal LAD due to the marked severity of the distal stenosis with predicted distal vessel FFR of 0.42 and only marginal increase in flow (3.1 to 3.7A).

## DISCUSSION

System equivalence is a term used in systems sciences to describe the notion that a component or parameter of a system behaves similar to a component or parameter of a different system such that the mathematical descriptions of these two systems are indistinguishable. Translational systems, such as mechanical, caloric, hydraulic and electrical can be readily shown to be equivalent.

The electronic-hydraulic analogy is the most widely used dynamical analogy for teaching the concepts of electron flow in basic physics classes. As electric current flow and other electronic processes are difficult to demonstrate, using their hydraulic equivalents in classrooms allows for easy demonstrations of the basic concepts of current flow and resistance. The electrical current (I) is equivalent to the fluid system’s volume flow rate (Q). The electrical driving voltage (V) is equivalent to the fluid driving pressure (P) or column height (h). Finally, electrical resistance (R) can be shown to be equivalent to a restrictive orifice within the hydraulic system.

Nonetheless, the situation is reversed in the case of computational modelling where electric models are more understandable and analyzable than fluid dynamic models. Most physicians are likely very familiar with and have a competent understanding of electric circuits as these are taught on most basic Physics classes, whereas true fluid dynamics models remain expensive, complex and within the purvue of more advanced college curricula. Consequently, the use of the hydraulic-electronic analogy to model fluid systems (in this case the coronary anatomy and epicardial stenoses) in the form of simple electric circuits can make the important concepts that underlie everyday clinical scenarios more easily accessible.

In this report, we present an interesting and complex case of serial epicardial vessel stenosis evaluation by fractional flow reserve in a patient with an occluded third coronary vessel supplied by collaterals.

The hemodynamic significance of each lesion is influenced by the presence of the other lesion that additionally limits maximal hyperemic flow through the artery. Consequently, the evaluation of the true hemodynamic significance by each of tandem epicardial lesions becomes an important limitation of standard FFR. Theoretical analysis and animal validation has shown that due to the interaction between serial stenoses, the usual FFR evaluation cannot be independently applied to each stenosis [19]. Instead, more complete equations are required as well as additional pressure measurements that necessitate balloon occlusion of the epicardial vessel and hence PTCA of at least one of the suspect stenoses.

The use of simplified electrical circuit models, as demonstrated herein, help demonstrate this interaction that exists between tandem resistors (viz. lesions). We encourage clinicians to experiment with the simple model, altering the resistance of each lesion such as to simulate PCI and thus gain a more insightful understanding into the interplay of reduced resistance and increased flow. It can also help explain why the elimination of resistance by stenting a distal diseased segment may, through increasing hyperemic flow, enhance the pressure gradient across a previously under-appreciated more proximal stenosis. The effect is most pronounced at a lesion supplying a large myocardial volume as the increased number of parallel microvascular resistors lead to high flow such that an angiographically “moderate” severity lesion can have a profound hemodynamic effect. This is evident in the present modelling as the proximal LAD lesion supplies both the LAD myocardium but also part of the RCA through collaterals such that the high flow leads to a significant pressure drop despite the moderate nature of the stenosis.

## CONCLUSION

Computation modelling and simulation of the cardiovascular system can help formulate hypotheses that may be tested by experimental studies. Similarly, experimental data can be used to inversely model algorithms for determining important cardiovascular parameters and thus aid in the construction of more accurate computational models. In the present article, we have demonstrated a further paradigm in computational modelling whereby easily accessible and simple cardiovascular models can be widely used, not by researchers, but rather by clinicians and educators as a tool to make complex clinical problems more accessible and facilitate the teaching and understanding of these concepts by trainee physicians.

## Figures and Tables

**Fig. (1):**
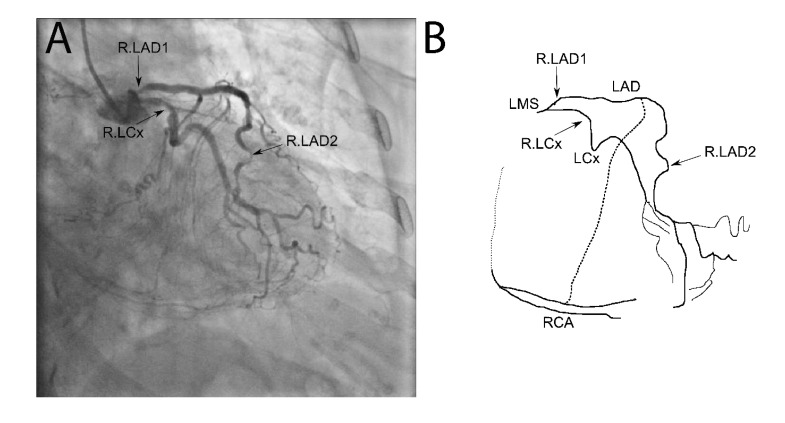
**Coronary angiogram and schematic.** The coronary angiogram is shown in panel A with the proximal LAD (R.LAD1), distal LAD (R.LAD2) and LCx (R.LCx) stenoses labeled. A schematic of the circulation is shown in panel 2. The collaterals from the mid LAD to the RCA are highlighted with a dark dashed line. The proximally occluded RCA course is shown with a light dotted line.

**Fig. (2):**
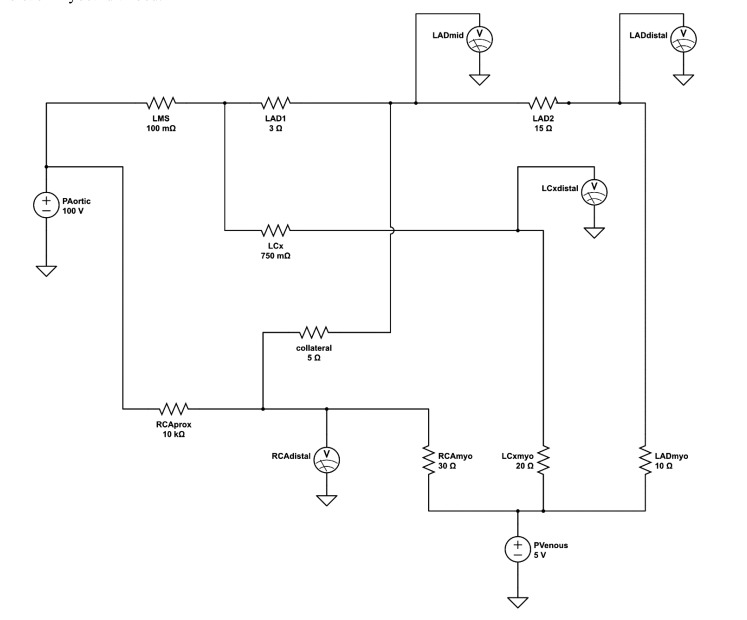
**Analog circuit coronary model.** Simplified analog electrical circuit to model coronary anatomy. Resistor LAD1 represents the proximal LAD stenosis while LAD2 represents the distal LAD stenosis at baseline values of 3Ω and 15Ω respectively. The mild LCx disease is modeled by resistor LCx (750mΩ). The proximal RCA resistance is high (10kΩ) to model the effective vessel occlusion. RCAmyo, LCxmyo, and LADmyo resistors represent the myocardial bed resistance of the RCA (30Ω), LCx (20Ω) and LAD (10Ω) territories respectively; current flow was measured across these resistors under different simulation settings. Voltmeters are positioned to measure voltages at the mid LAD, distal LAD, distal LCx, and distal RCA parts of the circuit.

**Fig. (3):**
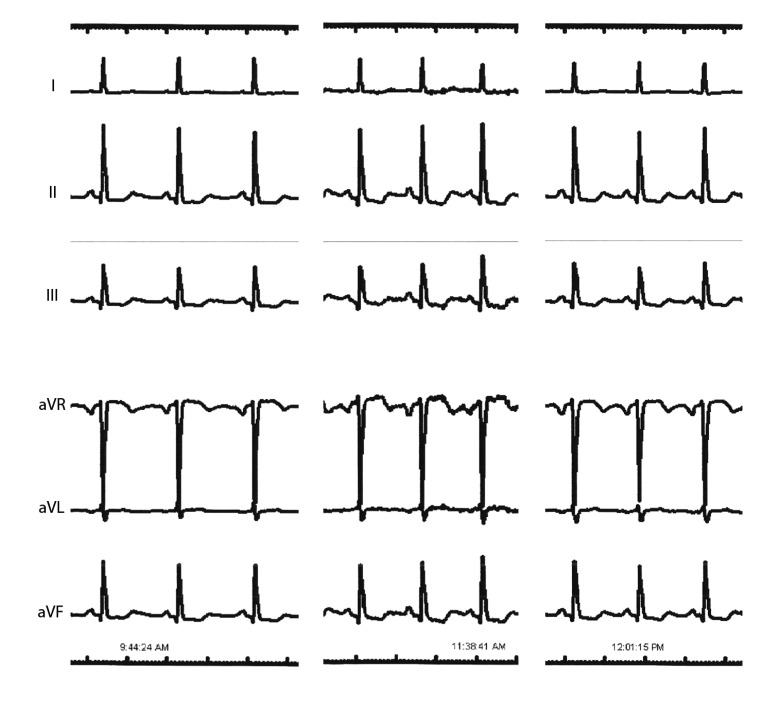
**ECG evidence of inferior wall ischemia after distal LAD PCI.** Following PCI to the distal LAD stenosis there is worsening ST depression in the inferior leads (II, III, aVF) that improves after further PCI to the proximal LAD stenosis.

**Table 1:** **Analog circuit simulation results.** LAD1 and LAD2 represent the proximal and distal LAD stenoses respectively. The voltage ratio is the voltage at each location noted divided by input voltage of 100V to simulate FFR. Simulation results shown include the baseline clinical state (sim 1), findings after distal LAD PCI (sim 2), and findings after further PCI to the proximal LAD (sim 3). In addition, a theoretical scenario of PCI to the proximal LAD stenosis without intervention to the distal LAD lesion is also modelled (sim 4).

	**Simulation 1**	**Simulation 2**	**Simulation 3**	**Simulation 4**
**Lesion Resistance (Ω)**				
LAD1	3	3	0.1	0.1
LAD2	15	0.1	0.1	15
**Voltage ratio**				
LAD mid	0.83	0.73	0.97	0.98
LAD distal	0.36	0.72	0.96	0.42
LCx distal	0.96	0.95	0.95	0.96
RCA distal	0.72	0.63	0.84	0.85
**Current Flow** **(A)**				
LAD	3.12	6.71	9.13	3.73
LCx	4.53	4.52	4.50	4.53
RCA	2.23	1.94	2.63	2.67
